# Effectiveness of Noise-Attenuating Headphones on Physiological Responses for Children With Autism Spectrum Disorders

**DOI:** 10.3389/fnint.2019.00065

**Published:** 2019-11-12

**Authors:** Beth Pfeiffer, Leah Stein Duker, AnnMarie Murphy, Chengshi Shui

**Affiliations:** ^1^Department of Health and Rehabilitation Sciences, Temple University, Philadelphia, PA, United States; ^2^USC Chan Division of Occupational Science and Occupational Therapy, University of Southern California, Los Angeles, CA, United States; ^3^School of Nursing, University of California, Los Angeles, Los Angeles, CA, United States

**Keywords:** hyperacusis, autism spectrum disorder (ASD), noise-attenuating headphones, noise canceling headphone, electrodermal responses (EDR), autonomic nervous system, stress, anxiety

## Abstract

**Objective**: The purpose of this study was to evaluate the proof of concept of an intervention to decrease sympathetic activation as measured by skin conductivity (electrodermal activity, EDA) in children with an autism spectrum disorder (ASD) and auditory hypersensitivity (hyperacusis). In addition, researchers examined if the intervention provided protection against the negative effects of decibel level of environmental noises on electrodermal measures between interventions. The feasibility of implementation and outcome measures within natural environments were evaluated.

**Method**: A single-subject multi-treatment design was used with six children, aged 8–16 years, with a form of Autism (i.e., Autism, PDD-NOS). Participants used in-ear (IE) and over-ear (OE) headphones for two randomly sequenced treatment phases. Each child completed four phases: (1) a week of baseline data collection; (2) a week of an intervention; (3) a week of no intervention; and (4) a week of the other intervention. Empatica E4 wristbands collected EDA data. Data was collected on 16–20 occasions per participant, with five measurements per phase.

**Results**: Separated tests for paired study phases suggested that regardless of intervention type, noise attenuating headphones led to a significance difference in both skin conductance levels (SCL) and frequency of non-specific conductance responses (NS-SCRs) between the baseline measurement and subsequent phases. Overall, SCL and NS-SCR frequency significantly decreased between baseline and the first intervention phase. A protective effect of the intervention was tested by collapsing intervention results into three phases. Slope correlation suggested constant SCL and NS-SCR frequency after initial use of the headphones regardless of the increase in environmental noises. A subsequent analysis of the quality of EDA data identified that later phases of data collection were associated with better data quality.

**Conclusion**: Many children with ASD have hypersensitivities to sound resulting in high levels of sympathetic nervous system reactivity, which is associated with problematic behaviors and distress. The findings of this study suggest that the use of noise attenuating headphones for individuals with ASD and hyperacusis may reduce sympathetic activation. Additionally, results suggest that the use of wearable sensors to collect physiological data in natural environments is feasible with established protocols and training procedures.

## Introduction

Unusual responses to sensory stimuli are experienced by up to 90% of individuals with autism spectrum disorder (ASD; Ben-Sasson et al., 2009). Although it is unclear as to whether sensory processing difficulties are a trait of ASD or a trait of comorbid disorders (Landon et al., 2016), behavioral responses to sensory stimuli have become so prevalent, that the most recent criteria in the Diagnostic and Statistical Manual of Mental Disorders 5th edition (DSM-V) for ASD added a diagnostic component of hyper- and hypo- reactivity to sensory stimuli (American Psychiatric Association, 2013). When studying the neurobiological differences in those with sensory difficulties, research indicates those with sensory over responsivity (SOR), or hypersensitivities, present with atypical sympathetic and parasympathetic functions of the nervous system (Miller et al., 2009). Of the various sensory responses, one of the most commonly reported challenges for those with ASD is hypersensitivity to sound (Baranek et al., 2006; Kern et al., 2006; Tomchek and Dunn, 2007; Stiegler and Davis, 2010; Bolton et al., 2012). Despite varying findings when analyzing cortical auditory sensory processing, neurophysiological studies have consistently identified atypical neural activity early in the processing stream in individuals with ASD (Marco et al., 2011).

Common in children with ASD, hyperacusis is a term used to describe the negative and/or exaggerated response to environmental stimuli occurring within the auditory pathways (Asha’ari et al., 2010; American Speech-Language-Hearing Association, 2016)^1^. Individuals with hyperacusis have an increased sensitivity to auditory input (Palumbo et al., 2018), and report experiencing auditory information at unbearably loud levels (Kuiper et al., 2019). Although hyperacusis is one of the most commonly identified auditory responses in children with ASD (Rogers et al., 2003), the cause of the disorder is not fully understood. Research suggests that the relationship between the central auditory system and the limbic system contribute to the development of the fear and anxiety frequently experienced with hyperacusis (Brout et al., 2018). In comparison to neurotypical peers, research on multi-sensory integration suggests that children with SOR may not process incoming information in lower level cortical regions. In conjunction with difficulties with sensory gating, challenges with modulation may prevent the central nervous system from appropriately identifying the intensity, frequency, duration, and complexity of environmental stimuli lending to issues filtering meaningful from non-meaningful sounds in the environment (Miller et al., 2009). This inability to filter may lead to an overwhelming amount of incoming stimuli, resulting in hyper-reactions due to sensory overload (Kuiper et al., 2019). The continual stress from perceived noxious stimuli and sensory overload can result in physiological changes (Rance et al., 2017). More specifically, decreased basal respiratory sinus arrhythmia and basal heart rate hyperarousal have been associated with social, language, and cognitive difficulties (Kushki et al., 2014).

Additionally, recent research has examined the role of medial olivocochlear efferent reflexes (MOC) in hyperacusis. Findings suggest that when comparing those with ASD with severe hyperacusis, those with ASD without hyperacusis, and neurotypicals, the MOC reflexes were twice as strong in individuals who have ASD with severe hyperacusis (Wilson et al., 2017). Despite this new understanding of the MOC reflexes and hyperacusis, research is inconsistent in identifying physiological differences in auditory pathways in individuals with hyperacusis (Tharpe et al., 2006; Jones et al., 2009).

Some emerging evidence suggests that SOR, such as hyperacusis, is associated with decreased inhibitory processes. For example, a fMRI study found slower habituation in youth with ASD and SOR in the amygdala and somatosensory cortex from both tactile and auditory input, as compared to youth with ASD without SOR (Green et al., 2015). Chang et al. (2012) found a significant association between electrodermal activity (EDA) and parent reported problem behaviors on the Sensory Processing Measure (SPM) Hearing and Total scale score categories. As discussed previously it is thought that hyperacusis may be linked to a difficulty in sensory modulation for children with ASD. Supporting this, Chang et al. (2012) found that participants with strong sympathetic reactivity were reported to have behaviors indicative of both over- and under-responsiveness. Through use of EDA, Schoen et al. (2008) also found two significant patterns of habituation in response to sensory stimuli (i.e., tone, strobe light, siren, smell, feather, chair movement). Within the population of children with ASD, their results grouped to show: (1) high tonic electrodermal arousal, high reactivity, and slower habituation; and (2) low tonic arousal, lower reactivity, and faster habituation.

When researching hyperacusis in adults with ASD, however, Kuiper et al. (2019) found no significant positive correlation between habituation rate and self-reported auditory hyper-sensitivity. Despite habituating at similar rates, it is noted that those with ASD had a higher skin conductance level (SCL) at baseline, indicating higher physiological arousal (Kuiper et al., 2019). Another study examined time-course responses of the auditory cortex to repeated auditory stimuli, as measured by magnetoencephalography, between boys with ASD who had auditory SOR, boys with ASD without auditory SOR, and neurotypical peers. The boys with ASD and auditory SOR exhibited prolonged response duration when compared to the other groups, suggesting decreased inhibition as found in abnormal sensory gating or dysfunction of inhibitory neurons (Matsuzaki et al., 2014). This was further supported in autism model rats that presented with a decrease in morphological size of the medial nucleus of the trapezoid body in the superior olivary complex, which holds an inhibitory role in auditory processing (Ida-Eto et al., 2017).

Regardless of the underlying cause, hyperacusis has been associated with anxiety and stress surrounding perceived noxious auditory stimuli, resulting in strong reactions (Jastreboff and Jastreboff, 2000; Brout et al., 2018). Illustrating this, children with ASD are frequently reported to cover their ears to block out sounds, as well as exhibit anxious or distressing reactions to some sounds (Rimland and Edelson, 1995; Jastreboff and Jastreboff, 2000). Intense and atypical responses to auditory stimuli can result in increased stress; avoidance of certain environments and interactions; decreased participation or engagement in key life activities and events; and distractibility impacting performance in home and school (Pfeiffer et al., 2019). These adverse effects on school performance and social interactions have been reported to influence overall quality of life (Grinker, 2007; Rowe et al., 2011; Smith and Riccomini, 2013). In a qualitative study by Landon et al. (2016), adult participants with ASD and noise sensitivity (NS) described particular sounds as causing physical discomfort and frustration. One participant with ASD and NS described “…the buzzing (of the fluorescent lightbulb) was so annoying that it got to the point where I couldn’t turn it on. So I sat there in the dark in my room for half the year because I couldn’t turn the light on (p. 48)”. Palumbo et al. (2018) note that characteristically, individuals with hyperacusis tend to become hyper-focused on listening for trigger sounds within their natural environments, resulting in a “perpetual state of anxiety” (p. 2) while they wait for the noxious stimuli to occur. This hyper-focused state was reported to cause emotional and physical discomfort by those with hyperacusis (Palumbo et al., 2018). In addition to experiencing emotional and physical discomfort, research indicates that chronic stress associated with hyperacusis may lead to negative mental and physical health conditions (McEwen and Gianaros, 2011).

Researchers have also examined the impact of noise on health in the general population. The World Health Organization (WHO) reports that environmental noise exposure can lead to a variety of negative health outcomes including sleep disturbance, cognitive impairments in children, stress-related mental health risks, as well as tinnitus (World Health Organization, 2018)^2^. Research suggests that autonomic nervous system and endocrine responses to sound correlate with particular night-time noises such as road traffic, aircrafts and railway noises, resulting in increased blood pressure, changes in heart rate, and leading to the release of stress hormones (Münzel et al., 2014). Research also suggests that individuals with non-supported coping strategies, such as children, may experience psychological stress in addition to physiological imbalance due to noise (Basner et al., 2014).

Due to the impact on participation and overall quality of life, a number of interventions that target the reduction of auditory hypersensitivity have been developed and trialed. One intervention, the listening project protocol (LPP), proposes to increase the neural tone to the inner ear muscles. During LPP, participants spend 45 min per day for 5 days listening to computer altered acoustic stimuli *via* headphones. This protocol was developed with insight from the Borg and Counter model, which suggests that auditory hypersensitivity in ASD may be due to atypical regulation of the middle ear as it tries to extract human speech from environmental noise (Porges et al., 2014). Research on this protocol showed a decrease in auditory hypersensitivity as well as an increase of spontaneous sharing behavior in children with ASD (Porges et al., 2014). However, several limitations were noted; for example, improvements were seen in both treatment and control groups, suggesting that the social engagement system utilized may have been a confounder. Although some research on this protocol has been conducted focusing on reducing auditory hypersensitivity in those with ASD, most of the clinical trials focus on its impact on emotional regulation and trauma^3^.

Another method currently utilized to reduce sound sensitivity in those with auditory hypersensitivities is auditory integration training (AIT; Sokhadze et al., 2016). Through filtered and modulated frequencies, AIT aims to suppress the peaks of frequency by random dampening of high and low frequencies in order to normalize the sounds and retrain the brain of someone who is hypersensitive (Sinha et al., 2006). Although different types of AIT, including the Listening Program, Berard Method and Tomatis Method are used, there is limited scientific research to support their ability to decrease auditory hypersensitivity (Dawson et al., 2007; Miller and Schoen, 2015; Sokhadze et al., 2016).

Researchers have also tested whether cognitive behavioral therapy (CBT) can alter learned patterns and behaviors, as well as faulty ways of thinking, related to particular noises in the environment. A randomized controlled trial was conducted in which a licensed psychologist trained in CBT provided six therapy sessions using CBT principles, psychoeducation, exposure therapy, applied relaxation and behavioral activation (Jüris et al., 2014). The use of CBT limits escape behavior by role playing potential problem scenarios (i.e., loud sounds or environments with unwanted noises) and learning to be calm (American Psychological Association, 2018). One of the benefits of using CBT for those with hyperacusis is the learning of new behaviors which can be used long after the study and intervention are completed (Jüris et al., 2014). The limitation of using CBT for those with ASD and hyperacusis, however, is that it requires recognition and awareness of the aversive stimuli. Event-related potential and magnetic field research on ASD (Orekhova and Stroganova, 2014) suggest that problems only arose when novel stimuli were outside of the individual’s focus of attention, suggesting CBT may be limited with those not aware of the actual trigger.

One common non-invasive intervention to improve the auditory environments for individuals with ASD are noise-attenuating headphones, which block sound transmission to the ears (Pfeiffer et al., 2019). Ikuta et al. (2016) conducted a pilot study on the effectiveness of noise-canceling (NC) headphones in children with ASD of varying intelligence. Participants in this study had difficulty using the NC headphones when they had hypersensitivity to human voices. As noted previously, one theory suggests that auditory hypersensitivity in those with ASD may be due to the inability of the middle ear to filter human voices from environmental noise (Porges et al., 2014). Research did find, however, that behavioral responses improved for children who perceived environmental noises (i.e., noisy classroom sounds) as noxious (Ikuta et al., 2016). Additionally, a single case design study identified an increase in attention to task for a child with ASD and auditory hypersensitivity when wearing the headphones (Rowe et al., 2011). Although this is often a low-cost and easily implemented intervention, there is limited research documenting its effectiveness. Additionally, to our knowledge, there is no current research examining the impact of environmental adaptation, such as use of noise-attenuating headphones, on the core issue of physiological anxiety and stress exhibited by individuals with ASD and hyperacusis.

Therefore, the purpose of this study was to examine the proof of concept for two types of noise attenuating headphones in reducing physiological stress and anxiety in children with ASD when in natural environments with noise perceived as aversive. Further investigation examined how the intervention provided a buffer for children with ASD against the negative effects of environmental noises on their physiological stress and anxiety. Historically, research assessing physiological responses to noise has been conducted in laboratory environments that does not reflect the natural environment. In children with ASD, participation in such studies do not accurately reflect the milieu of auditory stimuli encountered in the real world. For example, recent research has used fMRI to provide insight on neuronal correlations of auditory processing, although there is a loud noise associated with the imaging (Talavage et al., 2014) that is not typically encountered in natural environments. These imaging techniques, along with other psychophysiological measures such as EDA, require the participant to remain still for the duration of the recording (Boucsein et al., 2012; Boucsein, 2012; Wilson et al., 2017). Additionally, participating in these laboratory-based experiments may lead to increased stress as the child must deviate from his or her typical routine while in an unfamiliar environment with unfamiliar people. Because many children with ASD cannot express their distress in context-specific situations and their actions/reactions are often misunderstood, outcome measures of environmentally-based interventions in the natural context of the child are important to truly understand their experiences. Therefore, in this study, wearable sensors were used as the primary source of data collection with the intervention implemented in the natural environment. Due to complexities of collecting data within natural environments, we also evaluated the feasibility of the study measures and the quality of the wearable sensor data.

## Materials and Methods

### Design

The purpose of this study was to evaluate the proof of concept for an intervention to decrease physiological stress and anxiety among children with ASD within their natural environment. Single-subject multi-treatment design was used to compare two different noise attenuating headphone devices. These devices were over-ear (OE) BOSE Quiet Comfort 15 Acoustic Noise Attenuating Headphones and in-ear (IE) BOSE QuietComfort 20i Acoustic Noise Attenuating Headphones. The headphones were used to assess if noise attenuation would impact physiological responses during identified target activities with noxious auditory stimuli in natural environments. An ABAC design was used at random, assigning two different sequences of the intervention to the participants (Group A: ABAC or Group B: ACAB). Regardless of sequence, participants completed all four phases including: (1) a week of baseline data collection; (2) a week of an intervention; (3) a week of no intervention; and (4) a week of the other intervention. Participants did not wear the noise attenuating headphones during baseline or the week of non-intervention. During the 2 weeks of intervention, either the OE or IE attenuating headphones were used. Data collection occurred on 20 occasions per participant, with five measurements per phase. Participants were randomly assigned to one of two groups, with one group having phases sequenced ABAC and the other group having phases sequenced ACAB.

### Participants

In total, six children between the ages of 8 and 16 diagnosed with an ASD completed the study. Participant demographics are outlined in Table 1. Participants were only included in the study if they were diagnosed with a form of Autism using DSM-IV criteria. Diagnosis was confirmed through parent report and the completion of the Gilliam Autism Rating Scale 3rd edition (GARS-3; Gilliam, 2013)^4^. All participants had a score of 70 or higher, which is indicative of very high probability of ASD. Additionally, participants had to score in the probable or definite difference range on the Auditory Filtering and Auditory Sensitivity Scales of the Short Sensory Profile (SSP; Dunn, 1999) for inclusion as an indicator of hyperacusis.

**Table 1:** Participant characteristics.

Participant Number	Age	Diagnosis	Order of Intervention (B = OE; C = IE)	Score Range: Auditory Filtering (SSP)	Score Range: Visual/Auditory Sensitivity (SSP)	Activities and Environments Targeted for the NAH	Ethnicity of Child
3	8 years	PDD-NOS (DSM-IV); ADHD	ABAC	13/30 Definite Difference (range 6–19)	18/25 Probable Difference (range 15–18)	In car; Learning time with music	Caucasian
4	8 years	Autism (DSM-IV)	ACAB	14/30 Definite Difference (range 6–19)	18/25 Probable Difference (range 15–18)	Therapy session with music in background	Caucasian
8	8 years	Autism (DSM-IV)	ABAC	16/30 Definite Difference (range 6–19)	18/25 Probable Difference (range 15–18)	After school time; In the car	Caucasian
10	16 years	PDD-NOS (DSM-IV)	ABAC	15/30 Definite Difference (range 6–19)	14/25 Definite Difference (range 5–15)	Video games; Driving in car with music on; Grocery store; Homework	Caucasian
11	9 years	Autism (DSM-IV), ADD, SPD	ACAB	13/30 Definite Difference (range 6–19)	8/25 Definite Difference (range 5–15)	Playground; Occupational Therapy	Latin American or Hispanic
12	11 years	Asperger’s Disorder (DSM-IV), SPD, Anxiety	ABAC	16/30 Definite Difference (range 6–19)	15/25 Definite Difference (range 5–15)	Practicing drums; Playground	Latin American or Hispanic

Initially, 12 participants responded to recruitment efforts. However, six dropped out of the study before completing data collection. Reasons cited for participant drop-out included feeling overwhelmed or having problems with using technology; having no access to child during activities that were noisy and stimulating (i.e., school); confidentiality issues with conducting the experiment within the school setting; disruption of child’s routine; lack of time to do the data collection; constant monitoring of child to prevent destruction of headphones; and resistance of the child to wear the wristband collecting data.

### Procedure

Recruitment was conducted through social media, schools with individuals with ASD, private therapy practices, and community organizations in the Philadelphia area. Information about the study was posted on social media sites, such as Facebook. Flyers were provided to school administration, private therapy practices, and organizations that support individuals with ASD. If participants were interested, they contacted the research coordinator directly.

When an interested participant contacted the researchers, written informed consent was obtained from the parents of the participants who also completed a Demographic Questionnaire, GARS-3, and SSP to determine preliminary inclusion. If scores on the GARS-3 were 70 or higher, and scores on the Auditory Scales of the SSP fell in the range of probable to definite difference, a second meeting was scheduled to obtain child assent. All children provided assent through either verbal (i.e., verbal response of yes or no) or non-verbal indicators (i.e., nodding of head). Additionally, after having the child assent language read to them, the child signed an assent form if they were able. Once child assent was obtained, an occupational therapy evaluation was completed to identify activities/environments that were avoided or caused stress due to auditory stimuli, and to provide training on data collection methods. Target environments varied from child to child, including activities on the playground, playing with drums or video games, going grocery shopping, as well as other activities with and without music present (i.e., driving in a car, doing homework). During the study no was music played into the headphones so that the function was limited to providing noise attenuation rather than noise masking. Parents were provided with step-by-step training for use of equipment and other data collection procedures. See Table 2 for the Quick Reference Data Collection document provided to parents (the full Pictorial Direction Manual can be requested from authors).

**Table 2:** iPad with data plan instructions: procedures for BOSE data collection—quick reference.

	**Getting Started**
Step 1:	Put wristband on participant @ 20 min prior to start of activity, cover with sweatband.
Step 2:	Turn on iPod.
Step 3:	Press wristband power button for 2 s, it will blink green.
Step 4:	Open Empatica RT app.
Step 5:	Touch “start a new session,” then select Empatica E4 from
	device list.
Step 6:	Make a Visual Scan of environment.
Step 7:	Make a Decibel Reading of environment.
	**During Session**
Step 8:	Make a Visual Scan of Environment at middle and end of activity.
Step 9:	Make a Decibel Reading at middle and end of activity.
	**Ending Session**
Step 10:	Press the red X on Empatica RT App and confirm “ok?” to end data collection.
Step 11:	Remove wristband from participant and store in carrying case.
	**AFTER**
Step 14:	Open Notes App on iPod. Complete Momentary Assessment
	Questions.
Step 15:	Take screen shots of all decibel readings.
Step 16:	Close apps. Turn of iPod. Charge unit for next use.

Children had the opportunity to wear all study-related devices for a week before data collection began in order to help the child feel comfortable with the headphones as well as the wearable sensors within an environment/activity that was not targeted for the study. Participants who demonstrated refusal or discomfort after the one-week trial were excluded from the study. Throughout the study duration, a research team member checked in with participants regularly and provided technical support, as needed, throughout the data collection period. A gift card was given to the parent of a child who participated in the culmination of the study.

#### Intervention

Two different types of noise-attenuating headphones, designed to block out environmental noises, were used during the intervention phases. The technology used in these headphones compares and reacts to environmental sounds. When reacting to the environmental sound, a signal is provided to counteract the noise in the environment, thus canceling out the noise in the environment (BOSE, 2018)^5^. No music was played into the headphones during the study, so the headphones solely provided a noise attenuating function rather than noise masking. Although BOSE provided the equipment for the study, it is important to note that there are other organizations that produce headphone equipment that has noise attenuating features (e.g., QuietComfort, Velodyne, Etymotic and Westone).

Two types of noise attenuating headphones, IE and OE, were used during this study. The IE BOSE design used in this research has built-in “aware mode” technology. This allows the wearer to switch the processing applied to the microphones on the outside of each earbud creating an auditory approximation to removing the headphones (BOSE, 2018). In essence, the wearer could control how much of the environmental background noise they could hear or block out. The second, an OE device, did not have this mode and continually blocked noise in the environment. During the trial, the children/students wore these headphones during activities that had either large amounts of auditory stimuli or during activities that the child found aversive due to their perception of the auditory stimuli. Five points of data were collected in the 4 phases of: (1) a week of baseline data collection; (2) a week of an intervention; (3) a week of no intervention; and (4) a week of the other intervention.

EDA was collected using an Empatica E4 wireless wearable wristband device to measure arousal state. This wristband allows for researchers to receive data either in real-time or up to 60 h of data through a secure storage system (Empatica, 2018). For purposes of this study, the Empatica RT App was utilized to collect EDA data. Momentary assessment data was collected on types of daily activity, setting, and the number of people in the environment. This data was collected *via* Qualtrics on a provided iPod or iPad mini (Qualtrics, 2019)^6^. In addition, a visual scan of the environment was captured on the device’s camera application while in video mode to confirm reported data. For researchers to gain insight on actual environmental sound, smartphone technologies VenueDB app was used to collect decibel readings two times per session (EarMachine, 2018)^7^.

### Measures

#### Child Descriptor Measures

All parents of children participating in the study reported diagnosis based on the DSM-IV (i.e., PDD-NOS, Asperger Disorder, Autism), as their children were originally diagnosed using that classification system (American Psychiatric Association, 2000). Participant ASD diagnoses included Autism (*n* = 3), PDD-NOS (*n* = 2), and Asperger Disorder (*n* = 1). Additionally, ASD diagnosis was confirmed through completion of the GARS-3 (Gilliam, 2013). The GARS-3 is a widely used instrument to identify ASD and estimate its severity. A GARS-3 Autism Index score of ≥70 was used to confirm diagnosis (Gilliam, 2013).

The SSP (Dunn, 1999) was utilized to characterize auditory-specific sensory processing differences of study participants. This 38-item caregiver questionnaire is standardized for children ages 3–10 years. Using a five-point Likert scale, caregivers report how their child processes sensory information in day-to-day situations. On the *Auditory Filtering* subtests, all participants (*n* = 6) scored in the “definite difference” category, indicating difficulty filtering auditory stimuli in comparison to peers their age. On the *Visual/Auditory Sensitivity* subtest, three children scored “probable difference” and three children scored “definite difference.”

#### Outcome Variables

EDA reflects the skin conductance of the palmar sweat glands controlled by the sympathetic nervous system (Dawson et al., 2007), a marker of psychophysiological stress and anxiety. EDA was collected using the wireless Empatica E4, which was placed on the child’s non-dominant wrist and covered with a lightweight fabric band to ensure continuous contact between the electrode and the child’s wrist. The E4 utilized Ag/AgCl dry electrodes and sampled data at 8 Hz. Although the wrist is a non-traditional recording site, it has been found to be correlated with standard measurement locations (van Dooren et al., 2012) and previous research has utilized this equipment (or its predecessor, the Q-sensor) to collect EDA from the wrist in children with ASD (Baker et al., 2015, 2018, 2019; Fenning et al., 2017; Prince et al., 2017). EDA was recorded continuously throughout the study, beginning with a minimum of 20 min in order to allow sufficient buildup of moisture between the electrodes and the skin, followed by a baseline period and subsequent application of the experimental condition phase (baseline, IE, no intervention, OE). In longer-lasting situations, measurement of tonic SCL and frequency of non-specific skin conductance responses (NS-SCRs) are the most useful electrodermal measures (Dawson et al., 2007). It is well-documented that these tonic EDA readings increase in stressful or anxiety-producing situations (Dawson et al., 2007).

#### Covariates

Covariates were used to adjust for the estimation of the treatment effects including: presence of other people (1: 1–2 people; 2: 3–5 people; 3: 5–10 people; 4: 10–20 people, and 5: 20 + people); levels of visual stimuli (1: minimal, 2: moderate, and 3: a lot); levels of noise (1: quiet minimal, 2: moderate, and 3: a lot); setting (1: home, 2: community, and 3: school), and average value of the two decibel readings per session.

### Management of Electrodermal Activity (EDA) Data

For EDA data, the number of NS-SCRs were totaled for each participant and converted to a rate of fluctuations per minute and only counted when the amplitude was greater than or equal to 0.05 μs, as suggested by Dawson et al. (2007). Due to the skewed nature of our SCL and NS-SCR frequency data, and as is common practice with EDA data (Dawson et al., 2007), the Yeo-Johnson transformation (Yeo and Johnson, 2000) was applied to both SCL and NS-SCR frequency prior to modeling.

Data were visualized and downloaded in CSV format from the Empatica Connect Webportal for analysis. Data were imported into the BIOPAC program Acq*Knowledge* and a low-pass filter was applied to remove artifacts (Boucsein, 2012). Although ambulatory EDA datasets often preclude traditional quality assessment (e.g., “rigorous and methodical visual inspection and human coding”; Kleckner et al., 2018, pp. 1461; Boucsein, 2012; Boucsein et al., 2012), traditional visual inspection was possible due to the limited duration of each data recording in this study. Data cleaning was completed by hand, offline using Acq*Knowledge* to visualize the data in order to ensure deletion of movement artifacts (SCR data with a rise time <1 s, indicating an increase too quick to be attributable to physiological processes) and/or any abrupt drops which likely reflected the loss of contact between the skin and E4 electrodes. Both SCL and NS-SCRs were computer-scored off-line using the BIOPAC program Acq*Knowledge* and hand-checked to ensure no skin conductance responses were missed or incorrectly marked (Boucsein, 2012; Boucsein et al., 2012). Ten percent of the hand-coded data were double coded to ensure that the identification of NS-SCRs was reliable, with a minimum of 90% agreement (calculated as the number of matching NS-SCRs divided by the total number of NS-SCRs coded by the researchers). Overall, 88% of data were usable and included for analysis. Excluded data were flat line waveforms (SCL < 0.1 μs) with zero or few NS-SCRs. The unusable flat-lined data were due to equipment error/problems and not participants being electrodermal non-responders (Schoen et al., 2008; Keith et al., 2018), as other recordings from those participants yielded usable data. No data from one participant was initially usable; however, the participant collected a second round of data with 100% usability.

### Data Analysis

#### Analysis of Data Quality

To investigate how data quality varied systematically across study designs and under different conditions, we used a random effects ordered logistic model. Random effects models were chosen because the observations came from the same participants, violating the assumption of mutual independence. Ordered logistic models were utilized because the outcome variable (quality of data) had three ordered categories (1: not acceptable, 2: acceptable, and 3: highest quality). We regressed the outcome variable on selected variables, including the study design factors (intervention sequence: ABAC or ABAC; study phases: intervention or non-intervention) and covariates to identify potential associations. Finally, we conducted the Brant test to investigate the extent to which the ordered logistic model followed the assumption of parallel regression with this sample, which requires effects of the exploratory variables to be consistent across thresholds in the outcome variable. A cluster-robust estimator was used for statistical inferences.

#### Analysis of Preliminary Efficacy

A random-effects model, Moeyaert’s model parametrization (Manolov and Moeyaert, 2017), was used to evaluate the intervention’s treatment effects. Radom effects models account for auto-correlations among observations due to repeated measurements from the same participants, but can also take into consideration individual variations; for these reasons, random effects models are recommended when the main interest is to estimate the treatment effects of an intervention with repeated measurements (Manolov and Moeyaert, 2017).

This study adapts Moeyaert et al.’s (2014) multilevel models (Model 1A and 1B; p. 193). In these model specifications, the average treatment effects are captured by the mean values of the outcome variables among the observations during the specific study phases, adjusted for other covariates. We fitted two models with transformed NS-SCR frequency and transformed SCL as the outcomes and selected variables as the predictors (e.g., study design factors and covariates). We computed the adjusted average treatment effects using the fitted models and visualized the data to assist in clear interpretation. To determine the preliminary efficacy of the interventions, Wald-test was used to compare the adjusted average treatment effects between and across phases Although there were six completed cases with 108 observations, we limited our analysis to the acceptable data, yielding 95 observations across six participants. With this sample size, the model is limited regarding model complexity and numbers of independent variables. To better account for within-person correlations, we used robust estimator, maximum likelihood, and identity covariance structure in estimations. The unusable data (excluded and missing values) are of lower concern for this data as there were fewer than 12% of unusable values. Finally, we conducted statistical diagnosis and investigated the distributions of the residuals. Shapiro–Wilk normality test (W statistics) and Skewness/Kurtosis test ($\chi_{\left(2\right)}^{2}$) were applied to test for normality.

Additionally, we conducted supplementary testing to investigate potential mechanisms through which the interventions can effect SCL and NS-SCR frequency values. We hypothesized that the interventions could provide protection against the influence of decibel level of environmental noises on SCL and NS-SCR frequency. In other words, we sought to test the moderating effects of the interventions on the relationships between decibel levels and psychohysiological outcomes. To examine this, we completed the following three steps. First, the study phases were collapsed into three phases: (1) “No Intervention” (baseline + washout phases); (2) “In-Ear”; and (3) “Over-Ear.” Second, an additional interaction term between intervention and decibel reading was created and entered into the model to investigate the interaction effect. Third, a continuous time variable was created and entered into the model to adjust for potential time effects to account for the 4 week duration of the study. For these analyses, the random-effects model was used with robust estimator to handle clustering effects as we had to adjust for covariates, including noise levels, presence of other people, presence of visual stimulation, and activity types. All statistics were conducted in Stata 13.

## Results

### Data Quality Analysis

The ordered logistic model passed the Brant test ($\chi_{\left(11\right)}^{2}$ = 13.55, *p* = 0.259), suggesting that the model did not significantly violate the parallel regression assumption. As shown in Table 3, data quality was significantly associated with Phase 2 (first intervention in sequence), Phase 4 (second intervention sequence), and presence of other people. More specifically, the first intervention phase (Phase 2) was less likely to have higher quality data compared to baseline (Phase 1; 80% lower in odds; a.OR = 0.20, 95% CI: 0.06–0.65, *p* < 0.01). In contrast, the second intervention phase (Phase 4) was more likely to have higher quality data compared to baseline (197% higher in odds (a.OR = 2.97, 95% CI: 1.07–8.26, *p* < 0.05). Additionally, when an additional unit of people were present (e.g., 1: 1–2 people; 2: 3–5 people; 3: 5–10 people; etc.), the odds of obtaining higher data quality increased 82% (a.OR = 1.82, 95% CI: 1.48–2.25, *p* < 0.01). None of the other study design factors and covariates (e.g., activity type) significantly correlated with the data quality.

**Table 3:** Results of random-effect ordered logistic regression for data quality analysis.

	a.OR	SE	95% CI
Group B (vs. A)	1.49	0.27	(0.09, 25.99)
Study Phases			
Phase 2 (vs. 1)	0.20**	−2.70	(0.06, 0.65)
Phase 3 (vs. 1)	0.16	−0.80	(0.00, 14.27)
Phase 4 (vs. 1)	2.97*	2.09	(1.07, 8.26)
Noise Levels	1.25	0.24	(0.20, 7.92)
Presence of Other People	1.82**	5.64	(1.48, 2.25)
Presence of Visual Stimulation	0.50	−0.98	(0.13, 1.99)
Activity Types			
Stationary (vs. Active)	1.50	0.71	(0.49, 4.59)
Traveling (vs. Active)	3.24	1.21	(0.49, 21.57)
Average Decibel	1.03	1.50	(0.99, 1.08)
Time	1.09	0.40	(0.71, 1.69)

a.OR: adjusted Odds Ratio; *p < 0.05; **p < 0.01.

### Preliminary Intervention Efficacy

Results indicate that the residuals from both models followed normal distributions (for transformed NS-SCR frequency: *W* = 0.97931, *p* = 0.14687 and $\chi_{\left(2\right)}^{2}$ = 3.49, *p* = 0.1747; for transformed SCL: *W* = 0.99098, *p* = 0.78370 and $\chi_{\left(2\right)}^{2}$ = 0.71, *p* = 0.6997). Additionally, none of the predictors in the two models were significantly associated with the residuals. These results suggest that the risks of model misspecifications are low. The results of the model fitting are summarized in Table 4. For the transformed NS-SCR frequency, the main effect of Phase 2 (*β* = −0.58, 95% CI: −0.81 to −0.36, *p* < 0.01) and the interaction effect between Phase 2 and Group B (*β* = −0.74, 95% CI: −1.31 to −0.17, *p* < 0.05) were significantly associated with the outcome. In contrast, for transformed SCL, the interaction effect between Phase 2 and Group B (*β* = −1.16, 95% CI: −1.91 to −0.42, *p* < 0.01), as well as between Phase 4 and Group B (*β* = −1.19, 95% CI: −2.07 to −0.31, *p* < 0.01) were significantly associated with the outcome.

**Table 4:** Results of random-effect model for evaluation of intervention effects on electrodermal activity across study phases.

	Transformed NS-SCR Frequency			Transformed SCL		
	Beta	SE	95% CI	Beta	SE	95% CI
Group B (vs. A)	0.76	0.87	(−0.93, 2.46)	1.55^†^	0.80	(−0.02, 3.12)
Phase 2 (vs. 1)	−0.58**	0.11	(−0.81, −0.36)	−0.34	0.37	(−1.08, 0.39)
Phase 2 × Group B	−0.74*	0.29	(−1.31, −0.17)	−1.16**	0.38	(−1.91, −0.42)
Phase 3 (vs. 1)	0.35	0.66	(−0.95, 1.64)	0.06	0.22	(−0.37, 0.49)
Phase 3 × Group B	0.70	0.80	(−0.88, 2.27)	0.82	0.52	(−0.20, 1.84)
Phase 4 (vs. 1)	−0.03	0.58	(−1.15, 1.10)	0.32	0.41	(−0.48, 1.12)
Phase 4 × Group B	−0.88	0.68	(−2.22, 0.46)	−1.19**	0.45	(−2.07, −0.31)

^†^p < 0.1; *p < 0.05; **p < 0.01; the results were further controlled for noise levels, presence of other people, presence of visual stimulation, activity types, and average decibel.

**Figure 1:**
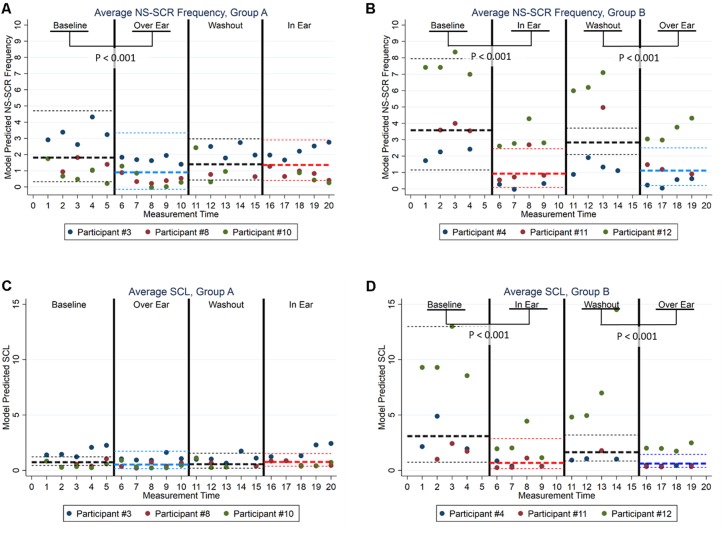
Model Adjusted Back-Transformed Outcomes by Electrodermal Activity (EDA), Study Designs, and Study Phases. **(A)** Group A Average NS-SCR. **(B)** Group B Average NS-SCR. **(C)** Group A Average SCL. **(D)** Group B Average SCL. *Note*. Bold broken lines represent the average values, with thin broken lines representing the 95% CI. Non-transformed values are presented here to increase interpretability; statistical tests were conducted using transformed data.

To assist in interpretation, we computed the model-adjusted average treatment effects across study groups and study phases, and applied Wald-test ($\chi_{\left(1\right)}^{2}$) to compare their average treatment effects (see Figures 1A–D). As illustrated in Figures 1A,B, Groups A and B followed similar patterns regarding participants’ psychophysiological responses to the interventions. More specifically, in Group A (Figure 1A), NS-SCR frequency was significantly lower in Phase 2 (Over Ear) in comparison with Phase 1 (Baseline; $\chi_{\left(1\right)}^{2}$ = 26.35, *p* < 0.001). Similarly, in Group B (Figure 1B), NS-SCR frequency was significantly lower in Phase 2 (In Ear) in comparison with Phase 1 (Baseline; $\chi_{\left(1\right)}^{2}$ = 42.75, *p* < 0.001), as well as in Phase 4 (Over Ear) in comparison with Phase 3 (Washout; $\chi_{\left(1\right)}^{2}$ = 12.76, *p* < 0.001). As illustrated in Figures 1C,D, we observed similar patterns in the SCL data. Although we did not find evidence of treatment effects in Group A for SCL scores (Figure 1C), in Group B (Figure 1D), the SCL scores were significantly lower in Phase 2 (In Ear) in comparison with Phase 1 (Baseline; $\chi_{\left(1\right)}^{2}$ = 53.72, *p* < 0.001), as well as in Phase 4 (Over Ear) in comparison with Phase 3 (Washout; $\chi_{\left(1\right)}^{2}$ = 54.72, *p* < 0.001).

Additionally, we investigated potential mechanisms in which the intervention may lower psychophysiological responses. We hypothesized that the intervention may provide protection such that when decibel values increased during the intervention phases both NS-SCR frequencies and SCL remained low. We fitted two random-effect models with an interaction effect between intervention phases and average decibel readings (see Table 5). Although we did not find evidence for the interaction effect between intervention and environmental decibel values for the transformed NS-SCR frequency, some significant relationships were found for the transformed SCL. Specifically, the interaction effects between decibel and In Ear (*β* = −0.02, 95% CI: −0.04 to −0.002, *p* < 0.05), as well as decibel and Over Ear (*β* = −0.02, 95% CI: −0.04 to −0.004, *p* < 0.05) were significantly associated with the transformed SCL scores.

**Table 5:** Results of random-effect models for moderation effects of intervention on the relationship between average decibel and electrodermal activity.

Model parameter	Transformed NS-SCR Frequency			Transformed SCL		
	Beta	SE	95% CI	Beta	SE	95% CI
Intervention					
In Ear (vs. No Intervention)	0.51	1.18	(−1.80, 2.81)	1.39	1.02	(−0.62, 3.40)
Average Decibel	0.02	0.01	(0.00, 0.04)	0.02*	0.01	(0.01, 0.04)
In Ear × Average Decibel	−0.01	0.01	(−0.04, 0.01)	−0.02*	0.01	(−0.04, −0.002)
Over Ear × Average Decibel	−0.01	0.01	(−0.03, 0.01)	−0.02*	0.01	(−0.04, −0.004)

*p < 0.05; the results were further controlled for noise levels, presence of other people, presence of visual stimulation, and activity types.

**Figure 2:**
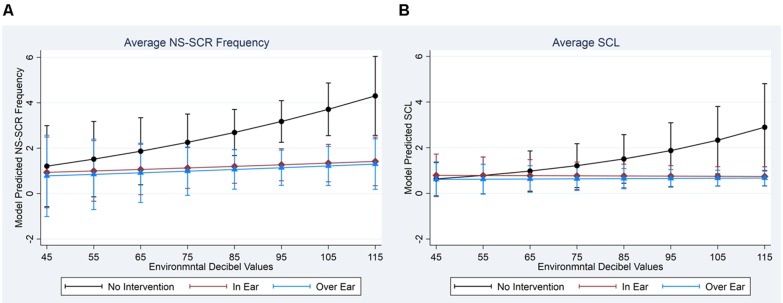
Model Adjusted Back-Transformed Outcomes by EDA and Intervention Types. **(A)** Average NS-SCR Frequency. **(B)** Average SCL. *Note*. Non-transformed values are presented here to increase interpretability; statistical tests were conducted using transformed data.

To assist in interpretation, we computed model-adjusted EDA measures across interventions and over the levels of environmental decibel readings (see Figures 2A,B for NS-SCR frequency and SCL, respectively). As illustrated in Figure 2A, when environmental decibel levels increased, NS-SCR frequency increased during the stages without intervention, while NS-SCR frequencies remained flat during the stages of interventions, although these differences did not reach statistical significance at 0.05 during formal testing (joint Wald-Test: $\chi_{\left(2\right)}^{2}$ = 2.09, *p* = 0.3524). Similarly, in Figure 2B, when environmental decibel levels increased, SCL increased during the stages without intervention but remained flat during the stages of intervention. These differences did reach statistical significance at 0.05 during formal testing (joint Wald-Test: $\chi_{\left(2\right)}^{2}$ = 8.07, *p* = 0.0177).

## Discussion

Research suggests that difficulties with auditory processing are more commonly reported than any other sensory disorder in individuals ASD (Tomchek and Dunn, 2007). Specifically, hyperacusis, a negative and/or exaggerated response to environmental stimuli related to auditory pathways (Asha’ari et al., 2010; American Speech-Language-Hearing Association, 2016), is one of the most identified characteristics of auditory processing differences. Researchers have identified that auditory dysfunction may be due to a slower auditory brain stem response in children with ASD (Lukose et al., 2013; Miron et al., 2018) and that there are anatomical links between the central nervous system and the amygdala (Myne and Kennedy, 2018) contributing to hyperacusis. Although hyperacusis is common in children with ASD, there is minimal scientific evidence to support commonly used interventions such as noise attenuating headphones, which reduces or blocks auditory stimuli in the environment. Results from the current study provide initial support for the use of noise attenuating headphones to reduce psychophysiological stress and anxiety from auditory stimuli, as measured by EDA. Additionally, results identified a clear positive relationship between the level of noise and EDA, which was buffered by the use of noise attenuating headphones.

Despite the neurological links identified between hyperacusis and ASD in research laboratory settings, to our knowledge, there is no research examining the impact of interventions in the natural environment on anxiety and stress levels within this population. As the greatest levels of over-responsiveness are found to be in multi-sensory environments full of potentially unknown experiences (Green et al., 2015), one limitation of laboratory research is that the testing environment does not reflect experiences that occur within natural settings. For example, a child with hyperacusis who has an aversive reaction to sirens on the highway may begin to associate the car with negative physiological experiences related to sounds that are found distressing. Subsequently, this may result in that child presenting with avoidance behaviors (i.e., tantrums, running away, crying) in anticipation of the sound when getting into or traveling in the car, even in the absence of the noise. As discussed previously, this has shown to increase stress for those who cannot communicate their feelings and can lead to the child being misunderstood.

Robertson and Simmons (2015) completed a focus group examining the sensory experiences of six adults with ASD. Results identified that all participants reported strong physical or emotional reactions to sensory stimuli in the environment. Lack of control over the sensory stimuli was identified as a factor that increased the perceived level of stress or anxiety. Prior to this, Smith and Sharp (2013) conducted a qualitative study in which adults with Asperger Syndrome reported sensory stress that contributed to strong emotional responses and coping strategies such as avoidance, fear and social isolation. Specific to the auditory sensory system, Landon et al. (2016) conducted qualitative research on adults with ASD and NS. Participants reported various ways in which hypersensitivity to noise impacted their participation in their day to day lives and the emotions experienced due to perceived noxious auditory stimuli. Despite some participants’ employing strategies such as the use of earplugs or verbally discussing their discomfort to sounds with those they knew, escaping from the potential problematic situation was common. Research indicates that a correlation exists among anxiety, SOR and behavior (Mazurek et al., 2013). Parents and families of children who are over-responsive to sensory stimuli often report avoiding events and activities due to the inability to prepare for potential unknown sensory experiences (Bagby et al., 2012; Demchick et al., 2014; Pfeiffer et al., 2017; Myne and Kennedy, 2018). A common natural context for children is school. Noting that the average noise level in classrooms exceeds WHO noise exposure guidelines, Keith et al. (2018) found that adolescents with ASD, as well as their matched neurotypical peers, performed worse on more difficult tasks when noise was added. Providing individuals with strategies to manage auditory hypersensitivities has the potential to aid them in participating in meaningful occupations rather than experiencing fear and anxiety and engaging in escape and elopement.

The current study employed the use of noise attenuating headphones within the natural environments of participants. On the basis of neural plasticity (Ayres, 1972) and experience-dependent plasticity (Alwis and Rajan, 2014), it is believed that active participation in enriched environments promotes neural change and cognitive behavioral improvements. Research has indicated that biochemical changes occur from engagement in meaningful trial and error learning during sensory and motor tasks (Miller et al., 2009). By decreasing anxiety and stress through the use of noise attenuating headphones, individuals can engage in this trial and error learning within their natural environment. It is further believed that repetition of normal responses to sensory stimuli creates new neural pathways thus providing the platform for successful participation in natural real-world environments (Miller et al., 2009). By providing a strategy that can be used on a day to day basis, individuals can develop the experiences and theoretically build the platform for successful participation. Though limited by small sample size and quasi-experimental design, past research implemented in natural environments identified positive behavioral and academic outcomes when using noise attenuating headphones in children with learning disabilities (Smith and Riccomini, 2013) and ASD (Rowe et al., 2011).

Additionally, the majority of evidence is founded in parent reports *via* questionnaires and interviews, as well as behavioral assessment of retrospective videotape analysis (Tomchek and Dunn, 2007; Myne and Kennedy, 2018). The wearable wireless technology used in the current study allows for the collection of physiological data measuring stress and anxiety within natural environments, creating a real-time picture of events and experiences of participants. As there are unpredictable environmental factors, it is important to consider their potential impact on data collection when using newer measurement systems such as wearable sensors. Analyses were completed for the current study on the quality of data collected from the wearable sensors. Results identified that quality increased over the course of data collection suggesting improvements with additional practice in using the technology. Since the data is collected in natural environments, it is often parents and caregivers who initiated data collection sessions. Although there was a high rate (88%) of useable data, additional practice sessions with the people who collect data may increase the overall quality when implementing research using wearable sensors. Additionally, there was an increase in the quality of data when more people were reported in the environment. It is possible that this resulted in more support for parents and caregivers from other people to ensure that data collection methods were properly implemented (i.e., assistance in maintaining the devices in proper position; ability to maintain focus on data collection methods), although this requires consideration in future research. Most importantly, results identified that neither the activity of the child nor the environmental setting had an impact on the quality of data suggesting that this type of data collection can be used across activity types.

In understanding the physiological responses in conjuction with the perceived experiences of parents/caregivers and individuals with ASD, we can develop and design more targeted interventions for auditory hypersensitivity. Psychologically, triggers may be more easily identified, and treatment/coping strategies can be assessed. If an individual can predict when they will be in an environment leading to this increased sympathetic activation, they may be able to use previously identified environmental interventions, such as noise attenuating headphones or other coping strategies, to continue participation rather than avoid engagement in important life activities and events. When triggered by stress, the emotional motor systems pathway activates one of the branches of the autonomic nervous system, the hypothalamic-pituitary-adrenal axis (HPA-axis; Mayer, 2000). The triggering of the emotional motor systems pathway can lead to emotional feelings and/or vigilance arousal, autonomic responses, sensory modulation and/or neuroendocrine responses. As interoceptive and exteroceptive stress responses occur, the cyclical effects of the triggering of the emotional motor systems pathways begin once again (Mayer, 2000). Thus, the use of noise attenuating headphones may decrease physiological responses in perceived auditory aversive situations, and may also provide opportunity for experiences as triggering of the HPA-axis may be avoided.

### Limitations

Similar to limitations of previous research on this topic, the study was limited in the sample size. This is due in part to the substantially varied environments within natural settings and the individualized nature of participants’ EDA that requires the use of single-subject design. Another limitation was the high dropout rate (*n* = 6; 50%). One suggested method to decrease drop-out rate would be to reduce the burden of data collection by using an automated measurement system that is activated at a designated decibel level, although this does not account for aversive responses to types of noises vs. levels of noise. In addition, no data was collected tracking the use of the aware mode during the IE headphone use. This feature could serve as a tool to design individualized interventions and should be explored in future studies.

### Future Research

Research has suggested that complete avoidance of sounds can lead to increased anxiety, therefore exacerbating the negative effects of hyperacusis (Jüris et al., 2014). Neurologically it is suggested that habituation-related plasticity occurs in the central limb of stress-response circuits allowing the hypothalamic pituitary adrenal axis to respond normally and possibly habituate to new environments (Day et al., 2009). Consistent with this neurological understanding, it has been shown that low levels of noise exposure may lead to desensitization of unwanted sounds (Jüris et al., 2014). Therefore, future research should implement methodology to track the use of the “aware-mode” for the IE headphones that allows the wearer to turn off the noise-blocking feature, enabling filtering rather than complete avoidance. This may prove more beneficial long-term in comparison to OE headphones without “aware mode.”

Although a link has been found between measures of EDA and parent-report, provider-report, and research-coded behavioral problems, it is highly recommended that future research incorporate behavioral measures of stress in order to examine whether a decrease in sympathetic activity has any relevant impact on child behavior and attention to task. Previous research has examined behavioral outcomes of using NC headphones but did not incorporate measures of sympathetic activity (Ikuta et al., 2016). Methodology could incorporate ecological momentary assessment to collect behavioral data in conjunction with wearable devices to examine the relationship between noise, sympathetic activity, and behavioral responses.

Additionally, there is a hypothesis in the literature that internal neuronal noise is a crucial factor influencing perceptual abilities in ASD. Emerging evidence suggests that high internal neuronal noise and poor external noise filtering impact auditory perception in individuals with ASD (Park et al., 2017). Recent research has implemented new measurement methods using EEG global coherence to examine the relationship between internal neuronal noise and the application of external auditory quasi-Brownian noise vs. absence of external noise (Mendez-Balbuena et al., 2018). Few studies have examined the relationship between EDA and EEG. Of those that have, correlations were found between SCL and specific EEG waveforms in girls with Attention-Deficit/Hyperactivity Disorder (Dupuy et al., 2014), as well as between EDA response amplitude during generalized tonic-clonic seizures and the duration of postictal generalized EEG suppressions in individuals with epilepsy (Poh et al., 2012; Onorati et al., 2017). In order to better understand the influence of auditory interventions, such as noise attenuating headphones, future research should examine the relationship between EDA and EEG global coherence in individuals with ASD during the presence and absence of targeted interventions. This would further expand the understanding of the relationship between internal neuronal noise and external noise filtering that is hypothesized to influence perceptual abilities.

## Ethics Statement

This study was approved by the Temple University IRB.

## Author Contributions

BP and LS contributed to the conception and design of the study. BP completed all the data collection and LS organized and interpreted all of the physiological data. CS assisted in data organization and analyzed the data. AM assisted in the organization of the data and data base, as well as helping in the writing of the introduction and discussion of the manuscript. BP (introduction, methodology and discussion), LS (parts of the methodology) and CS (data analysis and results) wrote the initial drafts of the manuscript. All authors contributed to manuscript revision, read and approved the submitted version.

## Conflict of Interest

The authors declare that the research was conducted in the absence of any commercial or financial relationships that could be construed as a potential conflict of interest.

## Footnotes

^1^https://www.asha.org/uploadedFiles/AIS-Hyperacusis.pdf

^2^http://www.who.int/sustainable-development/transport/health-risks/noise/en/

^3^https://integratedlistening.com/bing-safe-sound-protocol/

^4^https://www.pearsonassessments.com/store/usassessments/en/Store/Professional-Assessments/Behavior/Gilliam-Autism-Rating-Scale-%7C-Third-Edition/p/100000802.html?tab=product-details

^5^https://www.bose.com/en_us/products/headphones/noise_cancelling_headphones.html

^6^https://www.qualtrics.com

^7^https://www.earmachine.com/venuedb/
